# 5-fluorouracil treatment of patient-derived scaffolds from colorectal cancer reveal clinically critical information

**DOI:** 10.1186/s12967-022-03423-6

**Published:** 2022-05-13

**Authors:** Simona Salerno, Anders Ståhlberg, André Holdfeldt, Elinor Bexe Lindskog, Göran Landberg

**Affiliations:** 1 Department of Laboratory Medicine, Institute of Biomedicine, Sahlgrenska Center for Cancer Research, Sahlgrenska Academy at University of Gothenburg, Gothenburg, Sweden; 2 Wallenberg Centre for Molecular and Translational Medicine, University of Gothenburg, Gothenburg, Sweden; 3 Department of Clinical Genetics and Genomics, Region Västra Götaland, Sahlgrenska University Hospital, Gothenburg, Sweden; 4 Department of Surgery, Region Västra Götaland, Sahlgrenska University Hospital, Gothenburg, Sweden; 5 Department of Pathology, Region Västra Götaland, Sahlgrenska University Hospital, Gothenburg, Sweden; 6 Surgical Oncology Laboratory, Department of Surgery, Institute of Clinical Sciences, Sahlgrenska Academy at University of Gothenburg, Gothenburg, Sweden

**Keywords:** Colorectal cancer, Patient-derived scaffold, Decellularized matrix, 5-fluorouracil, Drug screening

## Abstract

**Background:**

Colorectal cancer is a commonly diagnosed cancer worldwide. Unfortunately, many patients do not respond to standard chemotherapy treatments and develop disease relapse and metastases. Besides cancer cell specific genetic changes, heterogeneity in the tumor microenvironment contribute to the clinical presentation of the disease and can potentially also influence drug resistance. By using a recently developed patient-derived scaffold method monitoring how a standardized reporter cancer cell line adapts to various microenvironments treated with chemotherapy, we wanted to clarify how individual patient specific microenvironments influence the chemotherapy response in colorectal cancer.

**Methods:**

Surgically resected colorectal cancer specimens from 89 patients were decellularized to produce patient-derived scaffold, which were seeded with HT29 cells, cultured for 3 weeks, and treated with 5-fluorouracil. Gene expression changes of adapted and treated HT29 cells were monitored by qPCR and compared with clinical parameters including disease-free survival.

**Results:**

The effects of 5-fluorouracil treatment varied between different patient-derived scaffold, but generally induced a reduced expression of proliferation genes and increased expression of pluripotency and epithelial-to-mesenchymal transition genes. Interestingly, patient-derived scaffold cultures obtained from patients with disease recurrences showed a significantly less pronounced anti-proliferative effect of 5-fluorouracil and more pronounced increase of pluripotency, with *MKI67* and *POU5F1* being among the most significant genes linked to disease relapse in colorectal cancer.

**Conclusions:**

Colorectal patient-derived scaffold can decode clinically relevant tumor microenvironmental influence of 5-fluorouracil treatment effects opening up for optimized precision medicine in colorectal cancer treatment.

**Supplementary Information:**

The online version contains supplementary material available at 10.1186/s12967-022-03423-6.

## Background

Colorectal cancer is the third most widespread type of cancer and the second most common cause of cancer-related death worldwide. Adjuvant chemotherapy is given to patients with high risk of recurrence [1] to ensure the suppression of cancer cells that may have remained or spread to distant organs. The most commonly used chemotherapy for colorectal cancer is 5-fluorouracil (5FU) [2] but recurrence after 5-FU therapy is unfortunately common and potentially linked to p53 mediated WNT/β-catenin signaling leading to cancer stem cell enrichment [3].

Although prognosis has improved for colorectal cancer, [4], it has been estimated that 30 to 50% of patients experience tumor relapse within 5 years [5]. Resistance or lack of response to chemotherapies is a major cause of failure of standard therapies and result in tumor relapse with spreading of cancer cells to distant organs [3, 6]. A large body of evidence exists showing that different components of the tumor microenvironment, including vasculature, stromal cells, signaling molecules and extracellular matrix (ECM), mediate the tumor response to treatments through various mechanisms [7–9].

Three-dimensional (3D) cell culture models are growing in popularity as drug screening platforms due to their ability to more accurately mimic physiological conditions compared to traditional 2D adherent cultures and have been valuable in early pre-clinical research for modeling complex mechanisms such as anticancer drug resistance [10].

We have recently developed an experimental patient-derived scaffold (PDS) model system and described how this 3D culture approach based on in vivo tumor material can recapitulate important patient specific clinical characteristics as relapse and cancer specific survival [11, 12]. The PDS model system consists of decellularized tumor samples including an imprint of important cancer progressing properties and events [13] that can be decoded by monitoring gene expression changes in an adapting standard cancer cell line [11, 12]. We have also demonstrated the advantage of the PDS model approach compared to 2D culture as a drug testing platform to monitor cellular responses to breast cancer chemotherapies and endocrine treatments in relation to the breast tumor microenvironment [14, 15].

The aim of this study was to extend the characterization of the PDS model approach to colorectal cancer and to evaluate the influence of colorectal tumor microenvironment on cellular response in relation to chemotherapy treatment. The colon cancer cell line HT29 was used as the adapter cells to monitor the 5FU treatment effects in PDS cultures from a large cohort of colorectal cancer patients with different clinical features. We have earlier shown that HT29 cells repopulated on PDS from colorectal tumors reveal important individual clinical information as cancer-specific survival and tumor location [12]. The results identified gene marker combinations affected by the tumor microenvironment and 5FU treatment linked to aggressive disease and high risk of relapse in colorectal cancer.

## Materials and methods

### Patient material and ethics statement

Colorectal tumor samples were collected at the time of surgery, snap-frozen in liquid nitrogen and stored at − 80 °C until use. Informed consent was obtained from all patients and the study was approved by the regional ethical review board in Gothenburg (DNR 118-15).

### Tumor decellularization

Tumor decellularization was performed as previously described [12]. Briefly, tumors were thawed at room temperature and cut in 5 × 5 × 5 mm pieces. Tumor pieces were then washed twice for 6 h in decellularization buffer (0.1% SDS (Sigma-Aldrich), 0.02% Na-Azide (VWR), 5 mM 2H_2_O-Na_2_-EDTA (Sigma-Aldrich), 0.4 mM phenylmethylsulfonyl fluoride (Sigma-Aldrich) in distilled water). After each wash, tumors were rinsed in the same buffer without SDS for 15 min. Decellularized tumors were then washed with distilled water for 72 h and finally with sterile PBS (Medicago) for 24 h. All wash steps were performed at 37 °C in a 10L Incu-shaker (Benchmark Scientific) with gentle shaking at 175 rpm. Finally, decellularized tumors (now considered PDSs) were sterilized in distilled water containing 0.1% peracetic acid (Sigma-Aldrich) for 1 h at room temperature and subsequently in PDS containing 1% Antibiotic–Antimycotic (ThermoFisher Scientific) for 24 h at 37 °C. PDS were stored at 4 °C in PBS containing 0.02% Na-Azide and 5 mM 2H_2_O-Na_2_-EDTA until subsequent use.

### 2D cell culturing

HT29 cells (ATCC HTB-38) were cultured and expanded in 2D conditions in McCoy´s 5A modified medium, supplemented with 10% fetal bovine serum and 1% penicillin/streptomycin (all ThermoFisher Scientific). Cells were passaged upon reaching 70–80% confluence.

### PDS culture

PDS were cut in 1 × 1 × 1 mm pieces and soaked in cell culture media for at least 1 h prior cell seeding to remove residual storage buffer. Cells were detached from plastic culture plates using trypsin (ThermoFisher Scientific) incubation for 2 min at 37 °C. Cells were then centrifuged for 3 min at 300 G, resuspended in medium and 5 × 10^5^ cells were added onto PDS in 1 mL cell medium in 48-well plates. Seeded PDS were incubated at 37 °C and not disturbed for 72 h. Subsequently and thereafter once a week, PDS were transferred to a new well with fresh medium. PDS were cultured for 21 days [12] prior drug treatment and downstream analysis.

### Matrigel culture

Matrigel (Growth factor reduced, ThermoFisher Scientific) was thawed on ice and diluted 1:2 with ice-cold medium. Then, 600 µL cold mixture was added to 24 well plates and incubated at 37 °C for 30 min to allow jellification. 3 × 10^5^ cells were then added on top of Matrigel in 1 mL cell medium. Matrigel culture were performed for 21 days, and medium was replaced every second day.

### 5-fluorouracil treatment

After 21 days of culture in either PDS or Matrigel, cells were exposed to 5FU (Teva, 50 mg/mL, purchased from Apoteket, Sweden) diluted in cell medium for 48 h.

### Cell viability assays

Cell metabolic activity following 5FU treatment was first determined in 2D-cultured cells via alamar blue assay (Invitrogen). Cells were seeded in a 96-well plate at a density of 6000 cells/well and cultured for 24 h. 10% alamar blue reagent was then added to the medium and incubated for 4 h at 37 °C. Alamar blue assay was repeated after a 48 h 5FU treatment with increasing concentrations (1 µM–200 mM). The post-treatment reading was normalized against the pre-treatment reading.

Cytotoxicity was determined in both 2D- and PDS-cultured cells by quantification of lactate dehydrogenase (LDH) in the conditioned medium following 5FU treatment, using Cytotoxicity Detection Kit (Roche).

### RNA extraction and quantitative polymerase chain reaction

2D- and PDS-cultured cells were washed twice with PBS, lysed in RLT buffer (Qiagen), snap-frozen and stored at − 80 °C. Matrigel samples were collected in 700 µL Qiazol (Qiagen), snap-frozen and stored at − 80 °C. Samples were then thawed on ice and homogenized using a stainless steel bead in TissueLyser II (Qiagen). RNA was extracted using RNeasy Micro Kit (Qiagen), including DNase treatment (Qiagen). RNA concentration was measured by NanoDrop (ThermoFisher Scientific).

Complementary DNA (cDNA) synthesis was performed using GrandScript cDNA synthesis kit (TATAA Biocenter). Reverse transcription of 100–500 ng RNA was performed on a T100 Thermal Cycler (BioRad) in 20 µl reactions at 22 °C for 5 min, 42 °C for 30 min, 85 °C for 5 min. The obtained cDNA was diluted 1:5 with water.

Quantitative polymerase chain reaction (qPCR) was performed on a CFX384 Touch Real-Time PCR Detection System (Biorad) using 6 µl reaction containing 1 × SYBR GrandMaster Mix (TATAA Biocenter), 400 nM of each primer (Sigma Aldrich, listed in Supplementary Table S1) and 2 µl diluted cDNA. The temperature profile was 95 °C for 2 min followed by 40 cycles of amplification at 95 °C for 5 s, 60 °C for 20 s and 70 °C for 20 s. Data pre-processing was performed using GenEx software (MultiD). All experiments were conducted in accordance with the Minimum Information for Publication of Quantitative Real-Time PCR Experiments (MIQE) guidelines [16].

### Data analysis and statistics

For experiments with n numbers lower than 9, statistical analysis was performed with Graphpad Prism (v9.0) using one-way analysis of variance with Dunnet’s post hoc test, unless otherwise stated. The statistical analysis of the large patient material data set (n = 89) was performed with SPSS Statistics (v25.0, IBM). Mann–Whitney U test was used to statistically compare two groups, Spearman’s rank correlation was used to determine paired associations in 5FU- treated and untreated PDS, and univariate Cox regression analysis was used to model disease free survival (DFS), defining events as tumor relapse or death by colorectal cancer.

## Results

### HT29 sensitivity to 5FU treatment in different cell culture conditions

**Fig. 1:**
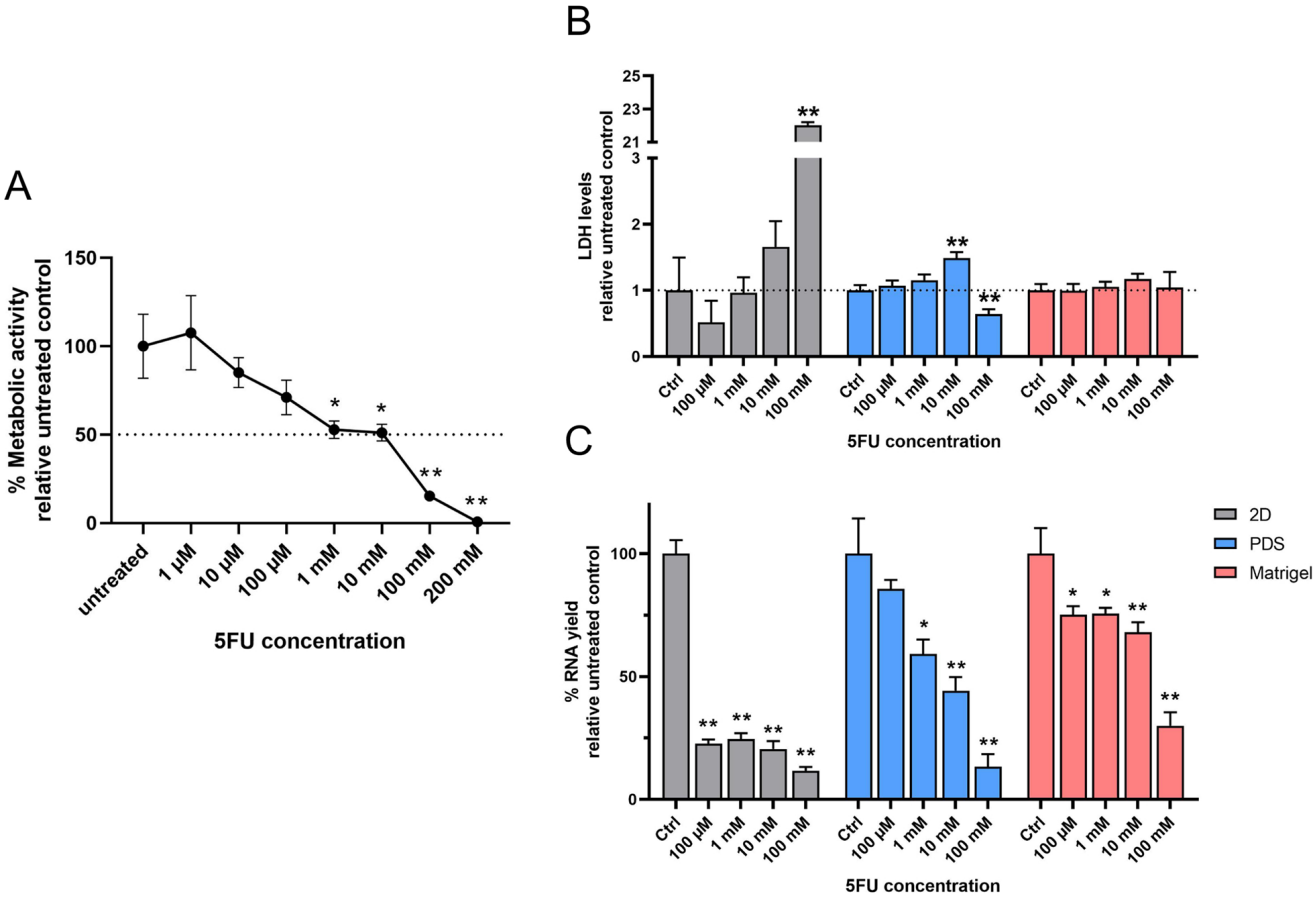
5FU treatment of HT29 cells cultured in 2D, PDS and Matrigel. **A** Percentage metabolic activity of 2D-cultured HT29 cells (n = 12) treated with 5FU relative to untreated cells. Mean ± SEM, *p < 0.05, **p < 0.01, one-way ANOVA with Dunnett’s post hoc test. **B** LDH levels of 5FU-treated HT29 cells cultured in 2D (n = 9), PDS (n = 9) and Matrigel (n = 5) relative to respective untreated controls. Mean ± SEM, *p < 0.05, **p < 0.01, one-way ANOVA with Dunnett’s post hoc test. **C** Percentage RNA yield of 5FU-treated HT29 cells cultured in 2D (n = 9), PDS (n = 9) and Matrigel (n = 5) relative to respective untreated controls. Mean ± SEM, *p < 0.05, **p < 0.01, one-way ANOVA with Dunnett’s post hoc test

To assess the sensitivity of the adapter cancer cell line HT29 to 5FU treatment independently from the influence of the surrounding environment, Alamar Blue assay was performed on 2D-cultured HT29 cells treated with increasing concentrations of 5FU. Metabolic activity of 2D-cultured HT29 cells was significantly reduced following a 48 h exposure to 5FU doses higher than 1 mM, and virtually absent with 200 mM 5FU (Fig. 1A).

Based on these results, we selected 5FU concentrations between 100 µM and 100 mM for further testing in 3D cultures. Since the Alamar Blue reagents were not suitable for 3D cultures, LDH activity assay was used to determine cytotoxicity. LDH levels of 5FU-treated 2D-cultured HT29 cells significantly increased with 100 mM 5FU, whereas cytotoxicity was detected in PDS-cultured cells when exposed to 10 mM 5FU. For Matrigel-cultured cells, no cytotoxicity was observed for any tested 5FU concentration (Fig. 1B).

In addition, RNA yield quantification was used as a surrogate measurement of cell numbers, which is affected by both proliferation effects as well as cell death. In 2D-cultured cells, RNA yields dropped significantly with all tested 5FU concentrations, reflecting a substantial decrease in cell numbers. The RNA yield of PDS-cultured cells decreased more gradually with increasing 5FU concentration and was significantly lower than untreated PDS-cultured cells for 5FU concentrations beyond 1 mM. For Matrigel-cultured cells, the RNA yield decreased significantly for all the 5FU concentration tested, but the drop in RNA yield was somewhat lower compared to 2D- and PDS-cultured cells. These results indicated that 3D-cultured cells, and in particular Matrigel-cultured cells, were less susceptible to 5FU treatment compared to 2D-cultured cells.

### Gene expression responses to 5FU treatment in different cell culture conditions

**Fig. 2:**
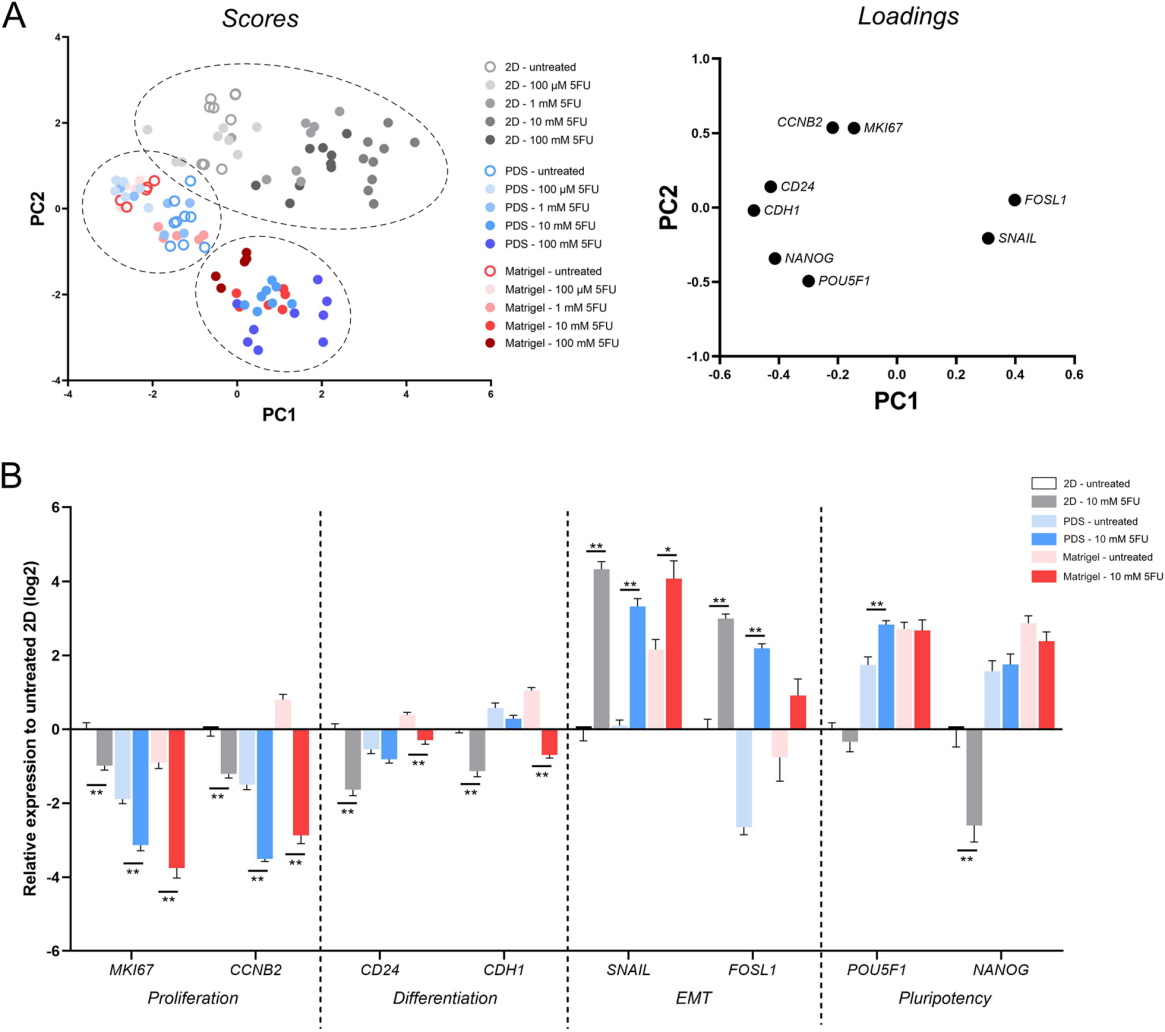
Genetic response of HT29 cells cultured in 2D, PDS and Matrigel to 5FU treatment. **A** Principal component analysis of gene expression data of HT29 cells cultured in 2D (grey symbols), PDS (blue sympols) and Matrigel (red symbols) and treated with increasing concentrations of 5FU. Data is expressed relative to untreated 2D-cultured HT29 cells. **B** qPCR data showing gene expression before and after 10 mM 5FU treatment in HT29 cells cultured in 2D, PDS and Matrigel. Data is expressed relative to untreated 2D cells. Bars represent Mean ± SEM, *p < 0.05, **p < 0.01, Student’s t test

Gene expression changes of 5FU-treated HT29 cells using different culturing conditions were assessed by qPCR. By using principal component analysis, overall similarities, and differences in 5FU response of 2D- and 3D-cultured cells could be visualized (Fig. 2A). A concentration-dependent clustering of 2D-cultured cells was observed and 10 mM 5FU induced the most pronounced gene expression response. In contrast, 3D-cultured cells separated from 2D-cultured cells and formed 2 distinct clusters. One of these clusters included all untreated 3D-cultured cells as well as 3D-cultured cells treated with 100 µM and 1 mM 5FU, whereas the other cluster included all 3D-cultured cells treated with 10–100 mM 5FU.

Although this indicated that 5FU treatment induced a similar gene expression response for 2D- and 3D-cultured cells, it should be noted that 3D scores never clustered together with 2D scores. Detailed bar plots for 10 mM 5FU treatment are illustrated in Fig. 2B, whereas the responses for different concentrations and models are presented in Additional file 1: Figure S1. A clear difference between the colorectal PDS and the other model systems was the 5FU-induced influence of the pluripotency genes *POU5F1* and *NANOG*, showing upregulation in PDS cultures, downregulation in 2D cultures and no differences in Matrigel cultures.

Based on cytotoxicity, RNA yield and gene expression data, a concentration of 10 mM 5FU was selected for the following extended PDS culture studies and all subsequent analysis.

### 5FU- induced gene expression fingerprint in colorectal PDS-cultured HT29 cells

**Fig. 3:**
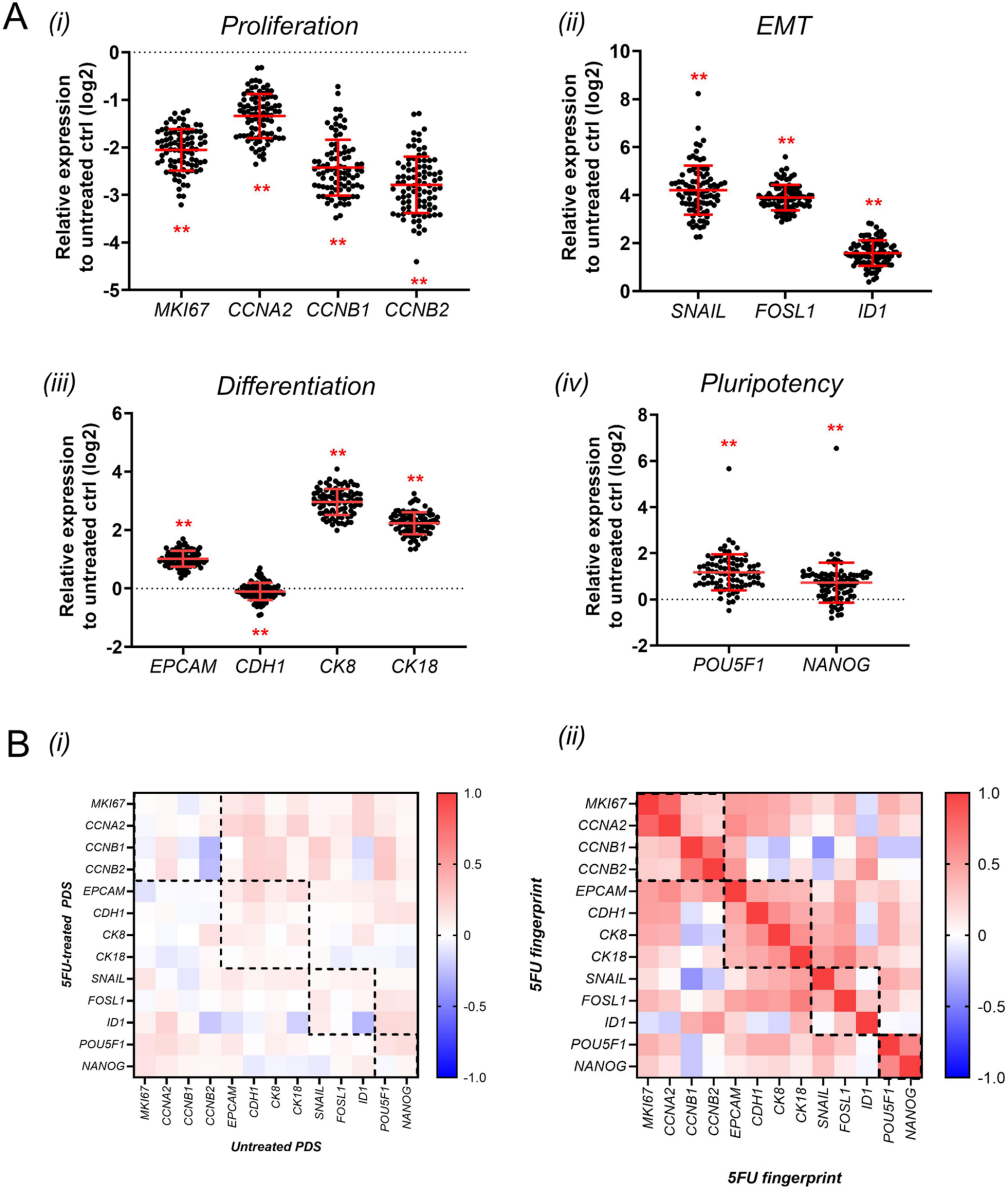
5FU treatment induces variable gene expression response in HT29 cells cultured in colorectal PDS. **A** 5FU-induced gene expression fingerprint. Dots represent 5FU response in individual PDS relative to the respective untreated PDS controls. Red bars indicate Mean ± SD (n = 89). *p < 0.05; **p < 0.01 (untreated PDS vs 5FU-treated PDS, Mann–Whitney U test). **B** Heatmaps representing Spearman’s rank correlation coefficients of (i) 5FU-induced gene expression fingerprint and (ii) between PDS- and 5FU-induced gene expression fingerprint

Next, we included 89 colorectal PDS with varying clinical characteristics to the treatment study (Additional file 1: Table S2). To isolate and exclusively study the 5FU effects on the adapter cancer cell line growing in the large set of individual PDSs, two PDSs were used for each patient. One PDS including cancer cells was left untreated and the other was exposed to 10 mM 5FU for 48 h after the initial 3 weeks culture period. Gene expression analysis was then performed and for each patient values for the untreated PDS were subtracted from the respective values for the treated PDS, creating a “fingerprint value” representing the 5FU effect in an individual tumor microenvironment. Figure 3A illustrates the resulting 5FU fingerprint data for all genes and individual PDSs. Results clearly showed that all proliferation markers were significantly downregulated by the 5FU treatment (Fig. 3A(i)) whereas all epithelial-mesenchymal transition (EMT) markers were upregulated (Fig. 3A(ii)). Differentiation markers were also upregulated after the treatment with the exception for CDH1 showing only minor changes (Fig. 3A(iii)). In addition, both pluripotency markers were significantly upregulated following 5FU treatment (Fig. 3A(iv)).

Next we investigated the Wnt/β-catenin pathway target genes, a signaling pathway implicated in relapse after 5-FU treatment [3]. Gene expression analysis showed significantly higher expression of Axin2, Lef1 and the p53 target gene CDKN1A (p21) markers in the PDS cultures compared to 2D cultures in HT-29 (Additional file 1: Figure S2A). However, for the “fingerprint values” representing the 5FU effect in an individual tumor microenvironment (comparing 5FU treated PDS to untreated PDS), Axin2 and LEF1 were significantly upregulated and downregulated respectively whereas the p53 target gene CDKN1A (p21) was significantly upregulated after 5-FU treatment. (Additional file 1: Figure S2B).

Besides the general trends described above, there was also clear variability between different PDSs supporting individual differences in the effect of the treatment in various cancer microenvironments.

Since we previously have shown that colorectal PDS cultures including the adapter cancer cell line HT29 resulted in variable gene expression adaptation [12], we compared the relation between the PDS induced changes and the additional treatment effects. Only minor correlations were observed between the gene expression measurements of treated and untreated PDSs (Fig. 3B(i)), suggesting that the 5FU treatment measurements produced additional information besides the direct scaffolds induced effects as summarized in Additional file 1: Tables S3A and S3B, respectively.

Next, Spearman’s rank correlation analysis was performed to identify possible associations among the genes that constitute the 5FU fingerprint (Fig. 3B(ii)). Interestingly, there was a significant positive correlation between genes belonging to the same category (highlighted with black dashed squares). This correlation was particularly strong (r_*S*_ > 0,6) between pluripotency genes and between subgroups of proliferation genes. A weaker correlation (0,4 < r_*S*_ < 0,6) was observed between differentiation genes and between two EMT genes (SNAIL and FOSL1). All data are summarized in Additional file 1: Tables S4A and S4B, respectively. Based on this observation, the gene expression values belonging to the same category was used to produce an average single category score for each tumor biological process and PDS.

### Significant association of 5FU-induced gene expression fingerprint of PDS-cultured HT29 adapter cells with patient clinical information

**Fig. 4:**
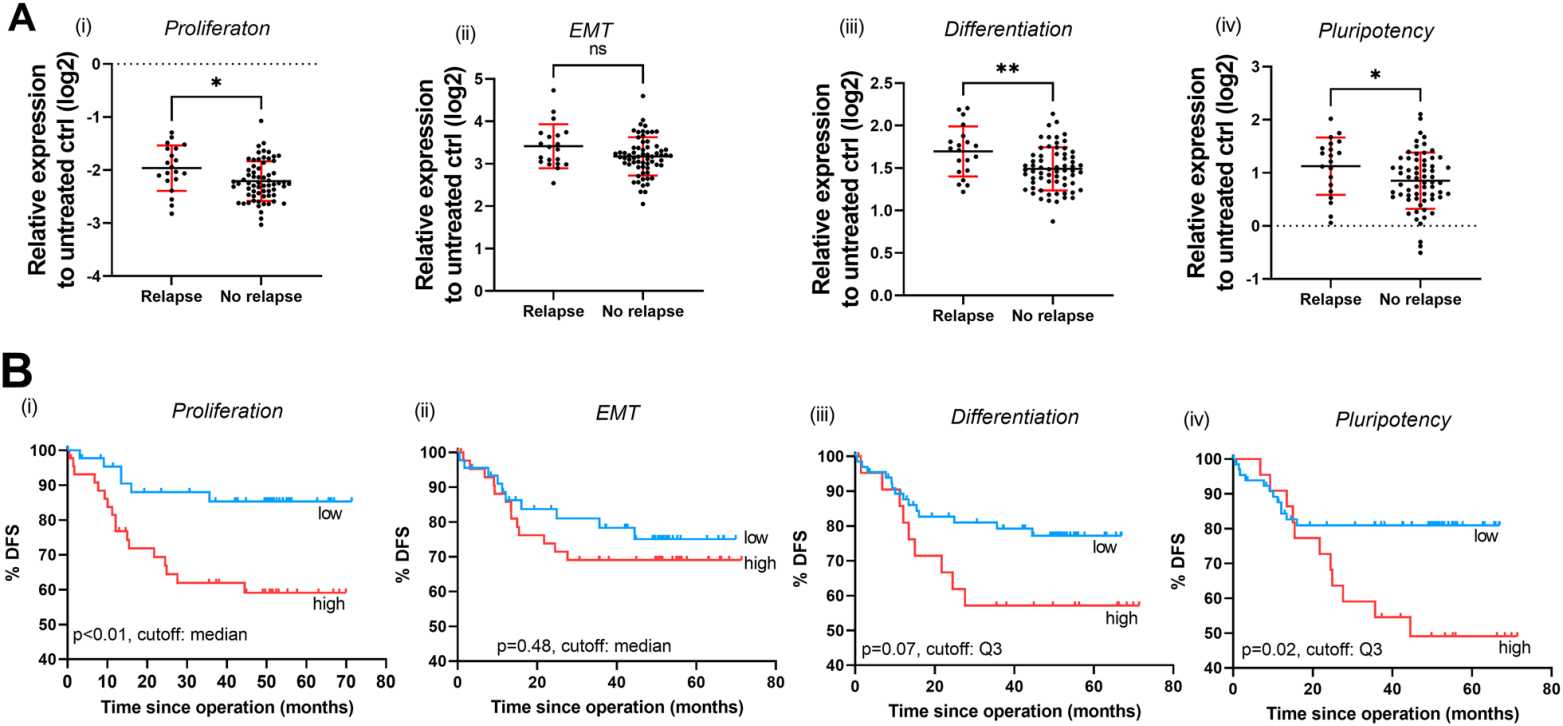
Associations of 5FU-induced expression of gene category scores with tumor relapse and disease-free survival. **A** Association of 5FU-induced expression of (i) proliferation, (ii) EMT, (iii) differentiation and (iv) pluripotency scores with tumor relapse in colorectal cancer patients. Gene category scores were calculated by averaging the expression of gene markers in individual patients. Proliferation category includes *MKI67*, *CCNA2*, *CCNB1*, *CCNB2*. EMT category includes *SNAIL*, *FOSL1*, *ID1*. Differentiation category includes *EPCAM*, *CDH1*, *CK8*, *CK18*. Pluripotency category includes *POU5F1*, *NANOG*. Mean ± SD (n = 65 no relapse, n = 20 relapse). *p < 0.05, **p < 0.01, Mann–Whitney U test. **B** Univariate Kaplan–Meier modeling of associations of 5FU-induced expression of (i) proliferation, (ii) EMT, (iii) differentiation and (iv) pluripotency scores with disease free survival (events are defined as relapse or death by colorectal cancer)

**Fig. 5:**
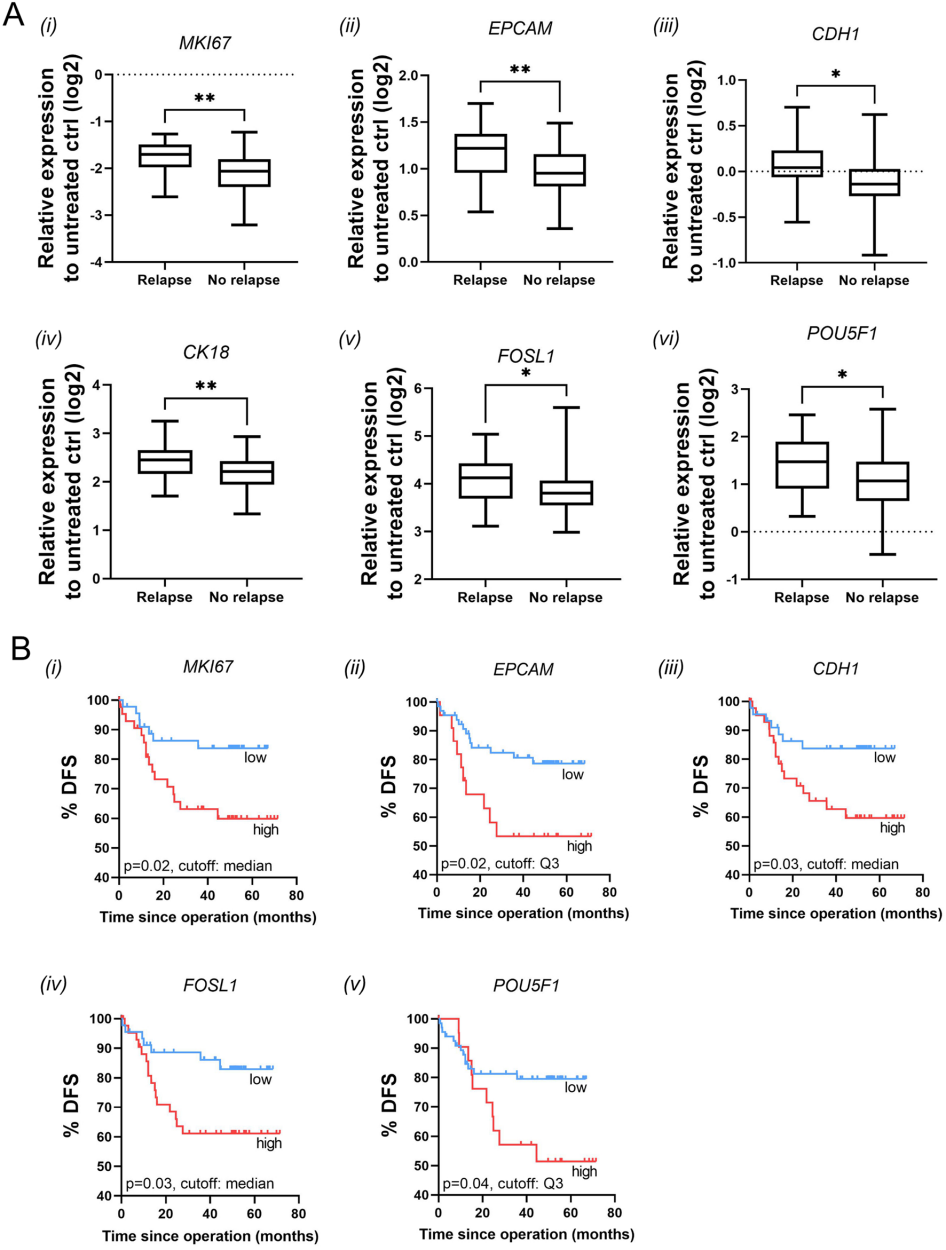
Associations of 5FU-induced expression of individual gene markers with tumor relapse and disease-free survival. **A** Associations of 5FU induced expression of (i) *MKI67*, (ii) *EPCAM*, (iii) *CDH1*, (iv) *CK18*, (v) *FOSL1* and (vi) *POU5F1* with tumor relapse in colorectal cancer patients. *p < 0.05, **p < 0.01, Mann–Whitney U test. **B** Univariate Kaplan–Meier modeling of associations of 5FU-induced expression of (i) *MKI67*, (ii) *EPCAM*, (iii) *CDH1*, (iv) *FOSL1* and (v) *POU5F1* with disease free survival (events are defined as relapse or death by colorectal cancer)

Nonparametric statistical analysis was used to clarify potential associations between clinical patient information and 5FU/PDS-induced gene category scores and single gene expression. Interestingly, disease relapse was significantly linked to proliferation as well as differentiation and pluripotency score changes. The 5FU-induced downregulation of proliferation genes was significantly more pronounced in PDS derived from patients who did not have disease recurrences (Fig. 4A(i)) and this was mainly governed by changes in *MKI67* (Fig. 5A(i)). Analogous, differentiation and pluripotency scores after 5FU treatment were significantly higher in PDS derived from patients with disease recurrences (Fig. 4A(iii–iv)). Within the differentiation category, *EPCAM*, *CDH1* and *CK18* were significantly associated with tumor relapse (Fig. 5A(ii–iv), whereas *POU5F1* was the major contributor within the pluripotency category (Fig. 5A(vi)). No significant association was found between EMT changes after treatment and patient relapse (Fig. 4A(ii)), although the expression of *FOSL1* was individually significantly associated with tumor relapse (Fig. 5A(v)).

Given the association with tumor relapse, the gene category scores were grouped into “high” and “low” 5FU response using either the median, the first quartile (Q1) or the third quartile (Q3) values as cutoff, and disease-free survival (DFS) analysis was performed using a univariate Cox proportional-hazard model. Events were defined as tumor relapse or death by colorectal cancer. The results clearly illustrated that a higher proliferation score as well as pluripotency score after treatment were strongly associated with an increased risk of tumor relapse or death by colorectal cancer (Fig. 4B(i) and B(iv), respectively). No significant association was found between EMT and Differentiation scores and DFS. When investigating individual genes, the most significant gene within the proliferation category score was *MKI67* (Fig. 5B(i)), while the most significant gene within the pluripotency family score was *POU5F1* (Fig. 5B(v)). In addition, differentiation gene markers *EPCAM* and *CDH1* and EMT gene marker *FOSL1* were also significantly associated with DFS (Fig. 5B(ii–iv)).

A complete and detailed univariate cox regression analysis of gene category scores and individual gene expression was summarized in Additional file 1: Table S5. Interestingly, the associations between gene expression in the PDS model and disease-free survival for the corresponding patients were only observed when using the 5FU fingerprint data and were not present before the addition of the treatment to the model (untreated PDS-data in Additional file 1: Table S6).

## Discussion

Resistance to cancer therapies is a major challenge for patients undergoing advanced therapies. Only recently has the tumor microenvironment been recognized as a potential critical modulator of therapy response. The tumor microenvironment varies between patients and has been shown to evolve and influence the course of the disease [17, 18]. Due to its critical role in cancer progression, it is important to include components of the tumor microenvironment into various pre-clinical tumor models to fully mimic mechanisms of drug resistance.

Transitioning from 2 to 3D cell culture model systems often confers increased resistance to cancer treatments as shown in simplified model systems such as tumor spheroids [19, 20]. Similar behaviors have been observed for more complex experimental systems incorporating components of the tumor microenvironment, including hydrogels resembling the ECM. In addition to providing structural support to the growing cells, complex model systems offer a more in vivo–like scaffold and framework for cell-ECM interactions. These models have improved our understanding of the tumor microenvironment on drug resistance [10]. For example, characteristics such as cell adhesion to the ECM, stiffness and collagen network clearly influence drug transport filtering into the tumor [21, 22]. However, these models cannot recapitulate the variation in tumor microenvironment observed between different cancer patients, and therefore cannot provide any clinically relevant information for the patients. In this context, our recently developed PDS model offers the advantage of transferring the clinical variability of the tumor microenvironment into a simple in vitro cell culture model. Published data also supports that the cell-free scaffold obtained in the PDS model system includes an imprint of important events in cancer progression including cues from different cell types and does not consist of only regular ECM proteins [13]. The possibility to decode this imprinted information via an adapter cell line that sense and adjust to the patient specific environment and then treat the “out-of-patient” model system with cancer drugs, will create a valuable surrogate system for modelling drug resistance and treatment prediction.

The observation that cancer cells cultured in colorectal PDS were less sensitive to 5FU treatment compared to standard adherent cultures are in line with earlier published data [14, 23]. Resistance to 5FU can develop through multiple and diverse mechanisms, including alterations of different pathways of 5FU metabolism [24] leading to perturbations of different cellular functions, such as apoptosis or cell cycle, with a significant regulatory role played by the tumor microenvironment [6]. In the present study, the reduced sensitivity to 5FU observed in PDS cultures did not seem to be related to reduced cell death, but rather to a reduced efficiency of 5FU in arresting cell cycle. This was clearly reflected in the quantification of RNA yield. Similar results have also been observed using the breast cancer cell line MCF7 cultured on breast tumor PDS following 5FU treatment [14].

In this study, we had the unique opportunity to evaluate the clinical relevance of colorectal PDS as a drug testing platform in a large cohort of colorectal patients with known clinical characteristics. Besides a general proliferation decrease for the adapter HT29 cells in PDS cultures compared to 2D cultures, 5FU treatment further decreased the expression of all proliferation markers analyzed. There was also a variation of this additive anti-proliferative effect of 5FU in PDS cultures, and a smaller 5FU-induced downregulation of proliferation in the PDS model was significantly associated with tumor relapse and DFS in the corresponding patients. High proliferation is often a sign of aggressive behaviors in cancer, and high expression of *MKI67* has been linked to worse DFS in cancer patients [25] [26–28].

The PDS model system can be used to monitor changes in various tumor relevant processes induced by scaffold growth and subsequent treatment. Besides proliferation, EMT and pluripotency are examples of key pathophysiological mechanisms that influence cancer progression, tumor relapse and spreading to distant organs [29, 30]. In this study, the expression of EMT and pluripotency markers was higher in 5FU-treated PDS compared to untreated PDS. This was probably due to of a 5FU-induced enrichment of cells with a stem cell-like and migratory phenotype which are notoriously resistant to chemotherapy treatment [31]. Interestingly, we observed a striking and significant link between a higher 5FU-induced pluripotency score and tumor relapse and DFS for patients. This means that if a treatment selectively spares and excludes cancer stem cells with pluripotency features due to the influences from the patient specific tumor microenvironment, the patient will most likely have a higher risk of recurrence despite treatment. The effect of the pluripotency expression score was mainly mediated by *POU5F1*. Interestingly, *POU5F1* has been associated with adverse prognostic features [32, 33] and chemoresistance [34] in colorectal cancer patients. 5FU-induced upregulation of POU5F1 was further only observed in colorectal PDS, and not in 2D cells or Matrigel cultures, supporting the clinical relevance of the PDS model.

Although the 5FU-induced EMT regulation in colorectal PDS was not significantly linked to disease recurrence, one gene marker within this category (*FOSL1*) correlated both with tumor relapse and DSF in patients. *FOSL1* encodes the FOS-related antigen 1 (FRA1), which is abnormally expressed in many types of tumors [35]. In colorectal cancer, *FOSL1* promote migration and invasion by maintaining cancer cells in a mesenchymal-like state [36, 37] and has further been linked to poor DFS [36].

Within the differentiation category, the expression of three gene markers (*EPCAM*, *CK8* and *CK18*) was increased in 5FU-treated PDS, while one (*CDH1*) was slightly but significantly reduced. In addition, three of these gene markers (*EPCAM*, *CDH1* and *CK18*) correlated with relapse and DFS in patients. The 5FU-induced regulations of this group of gene markers is also consistent with the observation that 5FU enriched for cells with aggressive features. In fact, upregulation of *EPCAM* [38–40] and *CK18* [41] as well as loss of *CDH1* [42, 43] have all been linked to aggressive and infiltrative tumors with poor sensitivity to drug treatment.

Within the WNT/β-catenin signaling pathway no genes tested were significantly linked to disease recurrence, however 5-FU treatment significantly increased Axin2 expression. Although Axin2 is considered as a tumor suppressor in colorectal cancer, increased expression has also been shown to induce EMT acting as a tumor promotor [44]. Interestingly, Lef1 that is as a nuclear effector in the Wnt/β-catenin signaling pathway was significantly downregulated after 5FU treatment, indicating a potential mechanism for 5FU effects. CDKN1A (p21) is a cell cycle inhibitor and is one of the target genes for p53 that is activated through Wnt/β-catenin signaling pathway cells. PUF family post-transcriptional regulators has also shown to promote colorectal cancer through suppression of CDKN1A (p21) [45]. 5FU-treament significantly increased CDKN1A (p21) expression, indicating a plausible mechanism for the anti-proliferate effects observed after the 5FU-treatment. The ambiguous role in colorectal cancer for Wnt/β-catenin signaling target genes has to be further investigated.

Both treatment resistance and 5FU-induced gene expression were similar in PDS and Matrigel. However, it is important to emphasize that, unlike PDS cultures, Matrigel cultures do not include materials derived from patients, and therefore have no clinical relevance linked to patients.

We have previously showed that the PDS model system influences gene expression of the cultured cell line, and that the varying effects from the patient specific tumor microenvironment was associated with patient clinical characteristics [12]. This clearly supports the concept that the qualities of the tumor microenvironment are linked to cancer progression. We have now added a new layer of characterization of the PDS model for colorectal cancer by showing that the response of an adapter cell line to chemotherapy treatment was influenced by the tumor microenvironment. Furthermore, the tumor microenvironment influenced the cancer cell adaptation to the PDS and the subsequent drug response independently as identified by the gene expression monitoring. In fact, the correlations with tumor relapse and DFS in this patient cohort were only observed when using 5FU fingerprint data [12]. Thus, with the PDS model system, it is possible to both delineate the effect of the tumor microenvironment on gene marker panels as well as the additive effect from chemotherapy treatment and the information can be relevant for various clinical challenges.

To our best knowledge, this is the first study reporting strong and significant associations between response to chemotherapy of an adapter cell line cultured on PDS model and DFS in the corresponding patients. There are nevertheless some limitations in the study design. The number of patients and amount cancer tissue available, only allowed testing of a single treatment option in a single cell line. 5FU was selected for this first pioneering study because it is the most common chemotherapy treatment for colorectal cancer. To increase treatment efficacy, 5FU is often administered in combination with Leucovorin and other chemotherapeutic agents, such as Oxaliplatin and Irinotecan [46]. Further studies using other chemotherapy regimens in the PDS model need to be performed to further elucidate mechanisms of multidrug resistance in relation to the tumor microenvironment and to evaluate the potential of the PDS model as a predictive tool to support clinicians with the identification of the optimal treatment for individual patients.

In the PDS model, the cell line is used as a “sensor” to monitor changes in 5FU responses influenced by the patient tumor microenvironment. By using a standardized adapter cancer cell line in the PDS-model instead of the patients own cancer cells, the success rate of the activity measurements of the microenvironment will be higher and the results more reliable by only varying the microenvironment and maintaining the adapting adapter cells identical and with the same genetic alterations. However, by including additional colon cancer cell lines from different molecular subtypes of the disease [47], the results can potentially be further refined even though the default cell line adapter approach used in this study indeed produce totally novel information highlighting the importance of the cancer microenvironment in malignant behaviours.

## Conclusions

This study has highlighted colorectal PDS as a clinically valuable and relevant drug screening platform that could monitor the influence of the tumor microenvironment in the response to 5FU treatment in colorectal cancer. This research and diagnostic tool also provided critical information regarding clinical aggressiveness for cancer patients representing a significant step forward in the field of personalized treatments.

## Supplementary Information

**Additional file 1:** **Figure S1.** 5FU dose response. **Figure S2.** WNT/β-catenin signaling pathway target genes. **Table S1.** Primer sequences for qPCR. **Table S2.** Clinical characteristics from consecutive patients operated for colorectal cancer. **Table S3A.** Spearman’s correlation coefficients of gene expression values in untreated and 5FU-treated PDS. **Table S3B.** p-values for Spearman’s correlation of gene expression in untreated and 5FU-treated PDS. **Table S4A.** Spearman’s correlation coefficients of 5FU fingerprint. **Table S4B.** p-values for Spearman’s correlation of 5FU gene expression fingerprint. **Table S5.** Univariate analysis modeling DFS using 5FU gene expression fingerprint data. **Table S6.** Univariate analysis modeling DFS using gene expression data in untreated PDS.

## Data Availability

The data used and/or analyzed during this study are available from the corresponding author on a reasonable request.

## Abbreviations

5FU: 5-Fluorouracil
ECM: Extracellular matrix
3D: Three-dimensional
PDS: Patient-derived scaffold
LDH: Lactate dehydrogenase
DFS: Disease free survival
EMT: Epithelial-mesenchymal transition

## References

[1] Argiles G, Tabernero J, Labianca R, Hochhauser D, Salazar R, Iveson T (2020). Localised colon cancer: ESMO clinical practice guidelines for diagnosis, treatment and follow-up. Ann Oncol.

[2] Longley DB, Harkin DP, Johnston PG (2003). 5-fluorouracil: mechanisms of action and clinical strategies. Nat Rev Cancer.

[3] Cho Y-H, Ro EJ, Yoon J-S, Mizutani T, Kang D-W, Park J-C (2020). 5-FU promotes stemness of colorectal cancer via p53-mediated WNT/β-catenin pathway activation. Nat Commun.

[4] Brenner H, Kloor M, Pox CP (2014). Colorectal cancer. Lancet.

[5] Guraya SY (2019). Pattern, stage, and time of recurrent colorectal cancer after curative surgery. Clin Colorectal Cancer.

[6] Blondy S, David V, Verdier M, Mathonnet M, Perraud A, Christou N (2020). 5-Fluorouracil resistance mechanisms in colorectal cancer: From classical pathways to promising processes. Cancer Sci.

[7] Wu T, Dai Y (2017). Tumor microenvironment and therapeutic response. Cancer Lett.

[8] Hirata E, Sahai E (2017). Tumor microenvironment and differential responses to therapy. Cold Spring Harb Perspect Med.

[9] Senthebane DA, Rowe A, Thomford NE, Shipanga H, Munro D, Mazeedi M (2017). The role of tumor microenvironment in chemoresistance: to survive, keep your enemies closer. Int J Mol Sci.

[10] Unger C, Kramer N, Walzl A, Scherzer M, Hengstschlager M, Dolznig H (2014). Modeling human carcinomas: physiologically relevant 3D models to improve anti-cancer drug development. Adv Drug Deliv Rev.

[11] Landberg G, Fitzpatrick P, Isakson P, Jonasson E, Karlsson J, Larsson E (2020). Patient-derived scaffolds uncover breast cancer promoting properties of the microenvironment. Biomaterials.

[12] Parkinson GT, Salerno S, Ranji P, Hakansson J, Bogestal Y, Wettergren Y (2021). Patient-derived scaffolds as a model of colorectal cancer. Cancer Med.

[13] Landberg G, Jonasson E, Gustafsson A, Fitzpatrick P, Isakson P, Karlsson J (2020). Characterization of cell-free breast cancer patient-derived scaffolds using liquid chromatography-mass spectrometry/mass spectrometry data and RNA sequencing data. Data Brief.

[14] Leiva MC, Garre E, Gustafsson A, Svanstrom A, Bogestal Y, Hakansson J (2021). Breast cancer patient-derived scaffolds as a tool to monitor chemotherapy responses in human tumor microenvironments. J Cell Physiol.

[15] Gustafsson A, Garre E, Leiva MC, Salerno S, Stahlberg A, Landberg G (2021). Patient-derived scaffolds as a drug-testing platform for endocrine therapies in breast cancer. Sci Rep.

[16] Bustin SA, Benes V, Garson JA, Hellemans J, Huggett J, Kubista M (2009). The MIQE guidelines: minimum information for publication of quantitative real-time PCR experiments. Clin Chem.

[17] Crotti S, Piccoli M, Rizzolio F, Giordano A, Nitti D, Agostini M (2017). Extracellular matrix and colorectal cancer: how surrounding microenvironment affects cancer cell behavior?. J Cell Physiol.

[18] Malik R, Lelkes PI, Cukierman E (2015). Biomechanical and biochemical remodeling of stromal extracellular matrix in cancer. Trends Biotechnol.

[19] Galateanu B, Hudita A, Negrei C, Ion RM, Costache M, Stan M (2016). Impact of multicellular tumor spheroids as an in vivolike tumor model on anticancer drug response. Int J Oncol.

[20] Daster S, Amatruda N, Calabrese D, Ivanek R, Turrini E, Droeser RA (2017). Induction of hypoxia and necrosis in multicellular tumor spheroids is associated with resistance to chemotherapy treatment. Oncotarget.

[21] Netti PA, Berk DA, Swartz MA, Grodzinsky AJ, Jain RK (2000). Role of extracellular matrix assembly in interstitial transport in solid tumors. Cancer Res.

[22] Grantab R, Sivananthan S, Tannock IF (2006). The penetration of anticancer drugs through tumor tissue as a function of cellular adhesion and packing density of tumor cells. Cancer Res.

[23] Sensi F, D'Angelo E, Piccoli M, Pavan P, Mastrotto F, Caliceti P (2020). Recellularized colorectal cancer patient-derived scaffolds as in vitro pre-clinical 3D model for drug screening. Cancers.

[24] Suetsugu T, Mori R, Futamura M, Fukada M, Tanaka H, Yasufuku I (2021). Mechanism of acquired 5FU resistance and strategy for overcoming 5FU resistance focusing on 5FU metabolism in colon cancer cell lines. Oncol Rep.

[25] Luo ZW, Zhu MG, Zhang ZQ, Ye FJ, Huang WH, Luo XZ (2019). Increased expression of Ki-67 is a poor prognostic marker for colorectal cancer patients: a meta analysis. BMC Cancer.

[26] de Azambuja E, Cardoso F, de Castro G, Colozz M, Mano MS, Durbecq V (2007). Ki-67 as prognostic marker in early breast cancer: a meta-analysis of published studies involving 12,155 patients. Br J Cancer.

[27] Pyo JS, Kang G, Sohn JH (2016). Ki-67 labeling index can be used as a prognostic marker in gastrointestinal stromal tumor: a systematic review and meta-analysis. Int J Biol Markers.

[28] Qiu D, Cai W, Zhang Z, Li H, Zhou D (2019). High Ki-67 expression is significantly associated with poor prognosis of ovarian cancer patients: evidence from a meta-analysis. Arch Gynecol Obstet.

[29] Cao H, Xu E, Liu H, Wan L, Lai M (2015). Epithelial-mesenchymal transition in colorectal cancer metastasis: a system review. Pathol Res Pract.

[30] Wahab SMR, Islam F, Gopalan V, Lam AK (2017). The identifications and clinical implications of cancer stem cells in colorectal cancer. Clin Colorectal Cancer.

[31] Dean M, Fojo T, Bates S (2005). Tumour stem cells and drug resistance. Nat Rev Cancer.

[32] Hu J, Li J, Yue X, Wang J, Liu J, Sun L (2017). Expression of the cancer stem cell markers ABCG2 and OCT-4 in right-sided colon cancer predicts recurrence and poor outcomes. Oncotarget.

[33] Miyoshi N, Fujino S, Ohue M, Yasui M, Takahashi Y, Sugimura K (2018). The POU5F1 gene expression in colorectal cancer: a novel prognostic marker. Surg Today.

[34] Wen K, Fu Z, Wu X, Feng J, Chen W, Qian J (2013). Oct-4 is required for an antiapoptotic behavior of chemoresistant colorectal cancer cells enriched for cancer stem cells: effects associated with STAT3/Survivin. Cancer Lett.

[35] Jiang X, Xie H, Dou Y, Yuan J, Zeng D, Xiao S (2020). Expression and function of FRA1 protein in tumors. Mol Biol Rep.

[36] Diesch J, Sanij E, Gilan O, Love C, Tran H, Fleming NI (2014). Widespread FRA1-dependent control of mesenchymal transdifferentiation programs in colorectal cancer cells. PLoS ONE.

[37] Liu H, Ren G, Wang T, Chen Y, Gong C, Bai Y (2015). Aberrantly expressed Fra-1 by IL-6/STAT3 transactivation promotes colorectal cancer aggressiveness through epithelial-mesenchymal transition. Carcinogenesis.

[38] Boesch M, Spizzo G, Seeber A (2018). Concise review: aggressive colorectal cancer: role of epithelial cell adhesion molecule in cancer stem cells and epithelial-to-mesenchymal transition. Stem Cells Transl Med.

[39] Seeber A, Untergasser G, Spizzo G, Terracciano L, Lugli A, Kasal A (2016). Predominant expression of truncated EpCAM is associated with a more aggressive phenotype and predicts poor overall survival in colorectal cancer. Int J Cancer.

[40] Spizzo G, Fong D, Wurm M, Ensinger C, Obrist P, Hofer C (2011). EpCAM expression in primary tumour tissues and metastases: an immunohistochemical analysis. J Clin Pathol.

[41] Zhang J, Hu S, Li Y (2019). KRT18 is correlated with the malignant status and acts as an oncogene in colorectal cancer. Biosci Rep.

[42] Chen X, Wang Y, Xia H, Wang Q, Jiang X, Lin Z (2012). Loss of E-cadherin promotes the growth, invasion and drug resistance of colorectal cancer cells and is associated with liver metastasis. Mol Biol Rep.

[43] Kim SA, Inamura K, Yamauchi M, Nishihara R, Mima K, Sukawa Y (2016). Loss of CDH1 (E-cadherin) expression is associated with infiltrative tumour growth and lymph node metastasis. Br J Cancer.

[44] Wu Z-Q, Brabletz T, Fearon E, Willis AL, Hu CY, Li X-Y (2012). Canonical Wnt suppressor, Axin2, promotes colon carcinoma oncogenic activity. Proc Natl Acad Sci.

[45] Gong Y, Liu Z, Yuan Y, Yang Z, Zhang J, Lu Q (2022). PUMILIO proteins promote colorectal cancer growth via suppressing p21. Nat Commun.

[46] Gustavsson B, Carlsson G, Machover D, Petrelli N, Roth A, Schmoll HJ (2015). A review of the evolution of systemic chemotherapy in the management of colorectal cancer. Clin Colorectal Cancer.

[47] Ahmed D, Eide PW, Eilertsen IA, Danielsen SA, Eknaes M, Hektoen M (2013). Epigenetic and genetic features of 24 colon cancer cell lines. Oncogenesis.

