# Supplementary material for: 5-fluorouracil treatment of patient-derived scaffolds from colorectal cancer reveal clinically critical information
Source: J Transl Med. 2022 May 13;20:209. doi: 10.1186/s12967-022-03423-6 (PMC9102939; doi:10.1186/s12967-022-03423-6)
Supplement: Supplementary file 1 — Additional file 1: Figure S1. 5FU dose response. Figure S2. WNT/β-catenin signaling pathway target genes. Table S1. Primer sequences for qPCR. Table S2. Clinical characteristics from consecutive patients operated for colorectal cancer. Table S3A. Spearman’s correlation coefficients of gene expression values in untreated and 5FU-treated PDS. Table S3B. p-values for Spearman’s correlation of gene expression in untreated and 5FU-treated PDS. Table S4A. Spearman’s correlation coefficients of 5FU fingerprint. Table S4B. p-values for Spearman’s correlation of 5FU gene expression fingerprint. Table S5. Univariate analysis modeling DFS using 5FU gene expression fingerprint data. Table S6. Univariate analysis modeling DFS using gene expression data in untreated PDS. [file 12967_2022_3423_MOESM1_ESM.docx]

# ADDITIONAL FILE 1 FIGURES


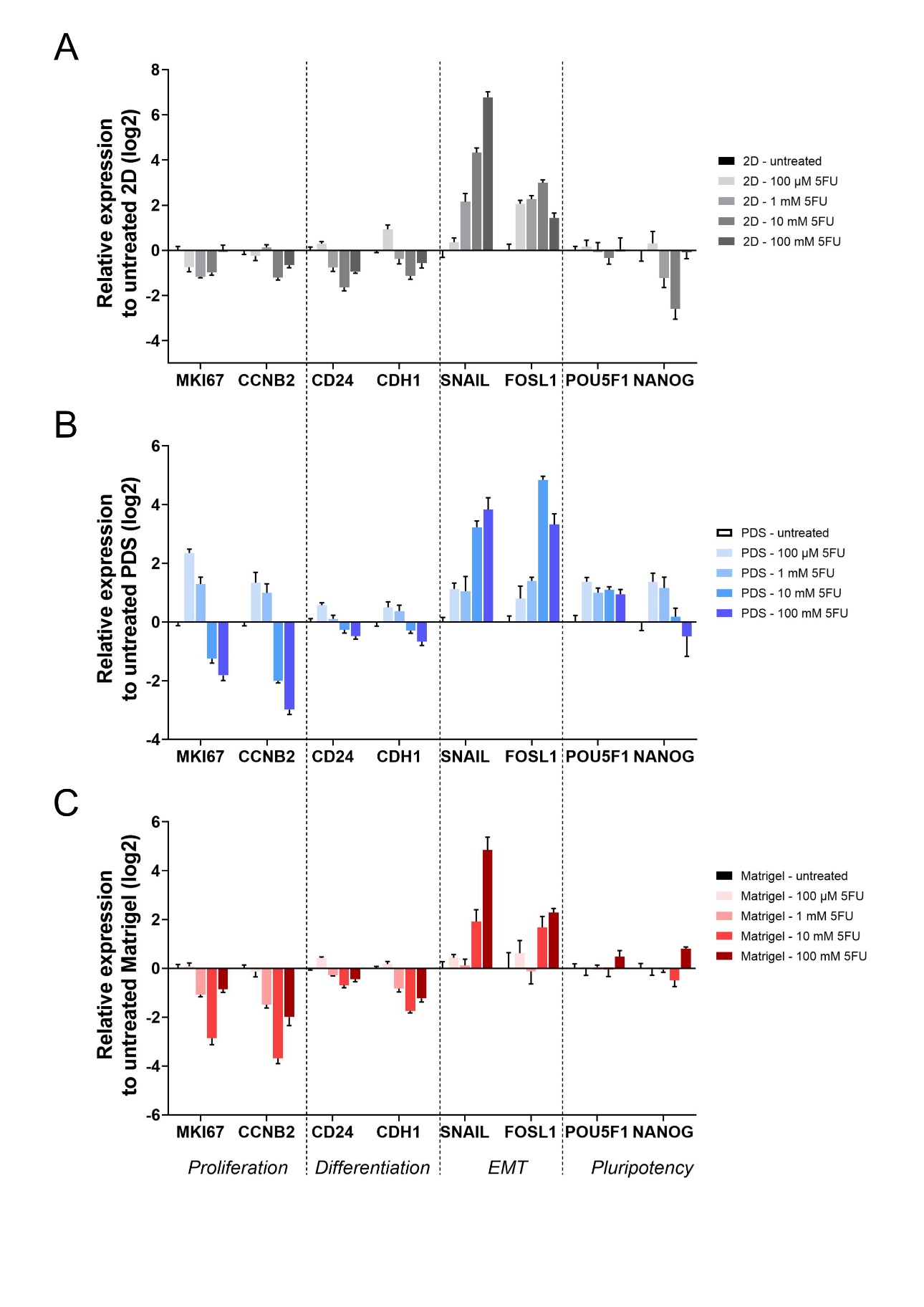


**Figure S1: 5FU dose response.** Genetic response to increasing 5FU concentration in **(A)** 2D, **(B)** PDS and **(C)** Matrigel cultures. Bars represent Mean ± SEM.


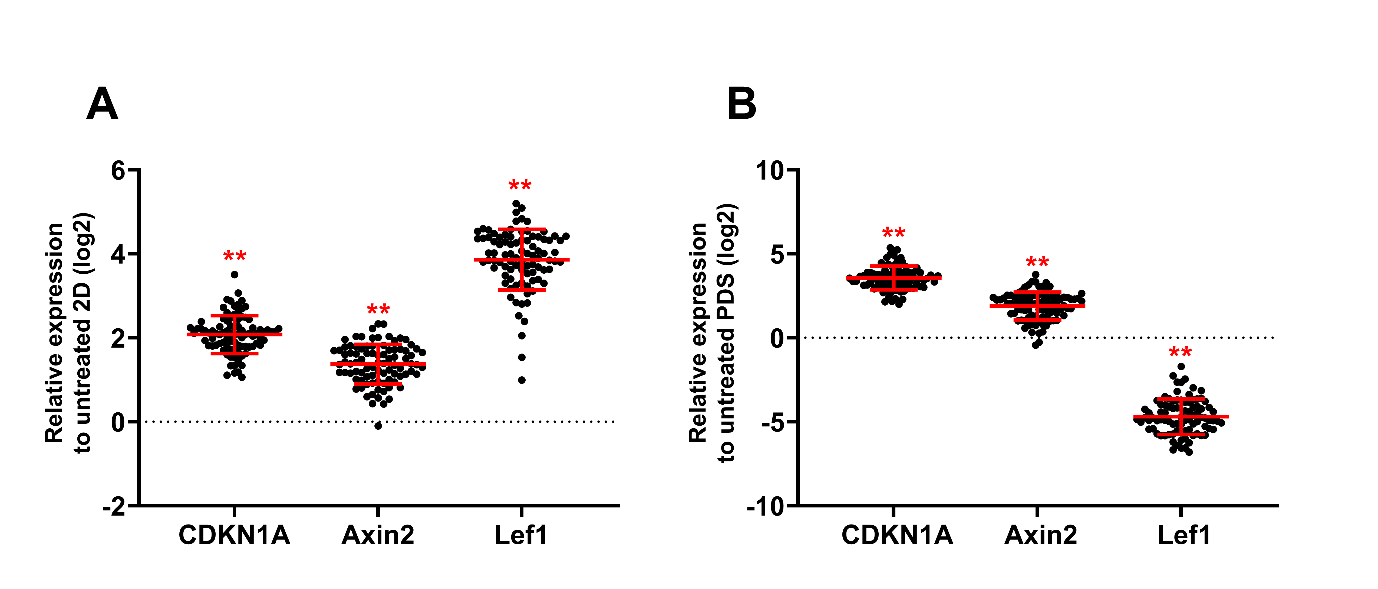


**Figure S2: WNT/ β-catenin signaling pathway target genes**. **(A)** Gene expression of HT-29 cells cultured in PDSs relative to the expression in two-dimensional (2D) cultures and expressed in log2-scale. Mean + SEM is shown, n = 89 PDS, n = 9 2D samples*p<0.05; **p<0,01 (untreated PDS vs 5FU-treated PDS, Mann-Whitney U test). **(B)** 5FU-induced gene expression fingerprint. Dots represent 5FU response in individual PDS relative to the respective untreated PDS controls. Red bars indicate Mean ± SD (n=89). *p<0.05; **p<0,01 (untreated PDS vs 5FU-treated PDS, Mann-Whitney U test).

# ADDITIONAL FILE 1 TABLES

| Gene | Forward sequence (5’-3’) | Reverse sequence (3’-5’) | Accession Number |
| --- | --- | --- | --- |
| *MKI67* | TGGGTCTGTTATTGATGAGCC | CATCAGGGTCAGAAGAGAAGC | NM_004360.4 |
| *CCNA2* | AAGACGAGACGGGTTGC | GGCTGTTTACTGTTTGCTTTCC | NM_001237.4 |
| *CCNB1* | TTCTGGATAATGGTGAATGGAC | ATGTGGCATACTTGTTCTTGAC | NM_031966.3 |
| *CCNB2* | CGACCCTTGCCACTACACTT | TGACTTCCAATACTTCATTCTCTG | NM_004701.3 |
| *SNAIL* | TAATCCAGAGTTTACCTTCCAGCA | AGCCTTTCCCACTGTCCTCA | NM_005985.3 |
| *FOSL1* | GCAGGCGGAGACTGACAA | GGGGAAAGGGAGATACAAGG | NM_001300855.2 |
| *ID1* | CTGAGGGAGAACAAGACCGAT | CCCCCTAAAGTCTCTGGTGA | NM_002165.4 |
| *EPCAM* | CAGGAAGAATGTGTCTGTGAAAACT | TTCATTTCTGCCTTCATCACC | NM_002354.2 |
| *CD24* | GCTCCTACCCACGCAGATT | GGTGGTGGCATTAGTTGGAT | NM_013230.2 |
| *CDH1* | AGAGGACCAGGACTTTGACTTG | CAGAGAATCATAAGGCGGGG | NM_004360.4 |
| *CK18* | CGAGAGACTGGAGCCATTACT | CGAGTCGTGTGATATTGGTGT | NM_000224.2 |
| *CK8* | CGACAAGGTAGAGCTGGAGTCT | CGAGCACCACAGATGTGTCCGA | NM_001256282.1 |
| *POU5F1* | CGAAAGAGAAAGCGAACCAG | AACCACACTCGGACCACATC | NM_002701 |
| *NANOG* | CCTATGCCTGTGATTTGTGG | AAGTGGGTTGTTTGCCTTTG | NM_024865 |
| *AXIN2* | GGTCCTGGCAACTCAGTAAC | AGTTCCTCTCAGCAATCGG | NM_004655.4 |
| *CDKN1A* | CAGGGGACAGCAGAGGAAGA | CGGCGTTTGGAGTGGTAGA | NM_000389.4 |
| *LEF1* | CCGTCACACATCCCATCAGA | GTTGCCTGAATCCACCCGT | NM_016269.5 |

**Table S1: Primer sequences for qPCR**

| Gender |  |  |
| --- | --- | --- |
|  | Male | 46 |
|  | Female | 43 |
| Age |  |  |
|  | Min | 40 |
|  | Max | 91 |
|  | Median | 71 |
| Tumor Location |  |  |
|  | Right-sided colon | 37 |
|  | Left-sided colon | 33 |
|  | Rectum | 19 |
| Relapse |  |  |
|  | Relapse | 24 |
|  | No relapse | 65 |
| Differentiation |  |  |
|  | Low | 13 |
|  | Medium | 64 |
|  | High | 6 |
|  | Mucinous | 6 |
| Stage |  |  |
|  | I | 16 |
|  | II | 35 |
|  | III | 26 |
|  | IV | 12 |

**Table S2: Clinical characteristics from consecutive patients operated for colorectal cancer**

|  |  | *untreated pds* | | | | | | | | | | | | | |
| --- | --- | --- | --- | --- | --- | --- | --- | --- | --- | --- | --- | --- | --- | --- | --- |
| **5FU-TREATED PDS** |  | | ***MKI67*** | ***CCNA2*** | ***CCNB1*** | ***CCNB2*** | ***EPCAM*** | ***CDH1*** | ***CK8*** | ***CK18*** | ***SNAIL*** | ***FOSL1*** | ***ID1*** | ***POU5F1*** | ***NANOG*** |
|  | ***MKI67*** | | *0,018* | *0,028* | *-0,078* | *0,037* | *0,118* | *0,175* | *0,027* | *0,122* | *0,046* | *0,042* | *0,225* | *0,082* | *0,043* |
|  | ***CCNA2*** | | *-0,035* | *0,039* | *0,013* | *0,08* | *0,208* | *0,257* | *0,084* | *0,232* | *0,055* | *0,1* | *0,222* | *0,114* | *0,034* |
|  | ***CCNB1*** | | *-0,054* | *0,135* | *-0,081* | *-0,284* | *-0,003* | *0,231* | *0,209* | *0,053* | *0,247* | *0,084* | *-0,143* | *0,285* | *0,066* |
|  | ***CCNB2*** | | *-0,011* | *0,173* | *-0,015* | *-0,259* | *0,062* | *0,236* | *0,22* | *0,051* | *0,205* | *0,038* | *-0,096* | *0,29* | *0,085* |
|  | ***EPCAM*** | | *-0,117* | *-0,019* | *-0,027* | *-0,019* | *0,108* | *0,225* | *0,095* | *0,174* | *0,058* | *0,069* | *0,09* | *0,126* | *0,038* |
|  | ***CDH1*** | | *0,015* | *0,028* | *-0,029* | *-0,034* | *0,04* | *0,123* | *0,057* | *0,026* | *0,012* | *0,017* | *0,051* | *0,132* | *0,143* |
|  | ***CK8*** | | *-0,003* | *-0,03* | *0* | *0,16* | *0,103* | *0,08* | *-0,008* | *0,099* | *0,013* | *-0,026* | *0,126* | *-0,021* | *-0,03* |
|  | ***CK18*** | | *-0,024* | *-0,091* | *-0,059* | *0,039* | *0,006* | *0,055* | *0,024* | *0,011* | *0,029* | *-0,073* | *-0,033* | *-0,036* | *-0,077* |
|  | ***SNAIL*** | | *0,135* | *-0,022* | *-0,098* | *0,054* | *0,121* | *0,062* | *0,09* | *0,102* | *0,03* | *0,065* | *0,078* | *0,031* | *0,035* |
|  | ***FOSL1*** | | *-0,029* | *0,043* | *-0,041* | *0,006* | *0,061* | *-0,007* | *0,036* | *-0,001* | *0,117* | *-0,013* | *0,045* | *0,132* | *0,121* |
|  | ***ID1*** | | *0,071* | *0,227* | *0,033* | *-0,217* | *-0,128* | *-0,05* | *0,066* | *-0,187* | *0,103* | *-0,008* | *-0,276* | *0,198* | *0,19* |
|  | ***POU5F1*** | | *0,162* | *0,185* | *0,112* | *0,053* | *0,123* | *0,015* | *0,081* | *0,044* | *0,004* | *0,046* | *0,015* | *0,144* | *0,181* |
|  | ***NANOG*** | | *0,152* | *0,099* | *0,049* | *0,041* | *0,05* | *-0,087* | *-0,041* | *-0,056* | *-0,084* | *0,03* | *0,101* | *-0,03* | *0,051* |

**Table S3A: Spearman’s correlation coefficients of gene expression values in untreated and 5FU-treated PDS.** Spearman´s correlation coefficients (r_S_) range between -1 and 1, indicating negative and positive correlation, respectively. │r_S_│> 0,6 is considered to be a “strong” correlation (**); 0,4 < │r_S_│< 0,6 is considered to be a “weak” correlation (*); │r_S_│< 0,4 is considered to be no correlation.

|  |  | *untreated pds* | | | | | | | | | | | | | |
| --- | --- | --- | --- | --- | --- | --- | --- | --- | --- | --- | --- | --- | --- | --- | --- |
| ***5FU-TREATED PDS*** |  | | ***MKI67*** | ***CCNA2*** | ***CCNB1*** | ***CCNB2*** | ***EPCAM*** | ***CDH1*** | ***CK8*** | ***CK18*** | ***SNAIL*** | ***FOSL1*** | ***ID1*** | ***POU5F1*** | ***NANOG*** |
|  | ***MKI67*** | | *0,871* | *0,794* | *0,468* | *0,73* | *0,271* | *0,1* | *0,803* | *0,254* | *0,667* | *0,698* | *0,034* | *0,443* | *0,689* |
|  | ***CCNA2*** | | *0,743* | *0,715* | *0,905* | *0,454* | *0,051* | *0,015* | *0,434* | *0,029* | *0,61* | *0,352* | *0,037* | *0,288* | *0,755* |
|  | ***CCNB1*** | | *0,613* | *0,206* | *0,448* | *0,007* | *0,977* | *0,029* | *0,05* | *0,623* | *0,019* | *0,431* | *0,18* | *0,007* | *0,536* |
|  | ***CCNB2*** | | *0,92* | *0,106* | *0,892* | *0,014* | *0,563* | *0,026* | *0,039* | *0,634* | *0,054* | *0,722* | *0,37* | *0,006* | *0,429* |
|  | ***EPCAM*** | | *0,275* | *0,862* | *0,799* | *0,859* | *0,316* | *0,034* | *0,376* | *0,103* | *0,587* | *0,519* | *0,4* | *0,241* | *0,727* |
|  | ***CDH1*** | | *0,886* | *0,798* | *0,787* | *0,752* | *0,709* | *0,252* | *0,593* | *0,812* | *0,913* | *0,877* | *0,637* | *0,219* | *0,181* |
|  | ***CK8*** | | *0,977* | *0,783* | *1* | *0,135* | *0,335* | *0,456* | *0,944* | *0,356* | *0,902* | *0,807* | *0,238* | *0,842* | *0,783* |
|  | ***CK18*** | | *0,824* | *0,394* | *0,584* | *0,714* | *0,955* | *0,611* | *0,822* | *0,916* | *0,789* | *0,496* | *0,76* | *0,736* | *0,476* |
|  | ***SNAIL*** | | *0,208* | *0,837* | *0,361* | *0,616* | *0,258* | *0,566* | *0,401* | *0,344* | *0,782* | *0,545* | *0,469* | *0,774* | *0,746* |
|  | ***FOSL1*** | | *0,789* | *0,692* | *0,706* | *0,957* | *0,572* | *0,945* | *0,734* | *0,996* | *0,276* | *0,902* | *0,677* | *0,218* | *0,26* |
|  | ***ID1*** | | *0,511* | *0,032* | *0,757* | *0,041* | *0,231* | *0,645* | *0,537* | *0,079* | *0,337* | *0,942* | *0,009* | *0,063* | *0,075* |
|  | ***POU5F1*** | | *0,155* | *0,355* | *0,65* | *0,704* | *0,641* | *0,417* | *0,703* | *0,599* | *0,436* | *0,778* | *0,346* | *0,781* | *0,632* |
|  | ***NANOG*** | | *0,129* | *0,082* | *0,294* | *0,623* | *0,251* | *0,891* | *0,453* | *0,685* | *0,967* | *0,668* | *0,89* | *0,179* | *0,089* |

**Table S3B: p-values for Spearman’s correlation of gene expression in untreated and 5FU-treated PDS.**

|  |  | *5FU fingerprint* | | | | | | | | | | | | | |
| --- | --- | --- | --- | --- | --- | --- | --- | --- | --- | --- | --- | --- | --- | --- | --- |
| ***5FU***  ***FINGERPRINT*** |  | | ***MKI67*** | ***CCNA2*** | ***CCNB1*** | ***CCNB2*** | ***EPCAM*** | ***CDH1*** | ***CK8*** | ***CK18*** | ***SNAIL*** | ***FOSL1*** | ***ID1*** | ***POU5F1*** | ***NANOG*** |
|  | ***MKI67*** | |  | ***0,805* | *0,276* | *0,251* | **0,505* | **0,497* | **0,431* | *0,290* | *0,180* | *0,379* | *-0,129* | **0,408* | *0,277* |
|  | ***CCNA2*** | | ***0,805* |  | *0,324* | *0,219* | **0,594* | **0,473* | **0,400* | *0,172* | *0,035* | *0,286* | *-0,193* | *0,295* | *0,143* |
|  | ***CCNB1*** | | *0,276* | *0,324* |  | ***0,696* | *0,396* | *-0,123* | *-0,244* | *0,005* | **-0,423* | *0,030* | **0,417* | *-0,219* | *-0,217* |
|  | ***CCNB2*** | | *0,251* | *0,219* | ***0,696* |  | **0,529* | *0,017* | *-0,164* | *0,158* | *-0,210* | *0,182* | **0,545* | *0,029* | *-0,017* |
|  | ***EPCAM*** | | **0,505* | **0,594* | *0,396* | **0,529* |  | **0,462* | *0,396* | *0,373* | *0,118* | **0,469* | *0,214* | *0,274* | *0,109* |
|  | ***CDH1*** | | **0,497* | **0,473* | *-0,123* | *0,017* | **0,462* |  | **0,585* | **0,446* | *0,355* | **0,538* | *-0,093* | **0,422* | *0,176* |
|  | ***CK8*** | | **0,431* | **0,400* | *-0,244* | *-0,164* | *0,396* | **0,585* |  | **0,572* | **0,420* | **0,494* | *-0,156* | **0,426* | *0,201* |
|  | ***CK18*** | | *0,290* | *0,172* | *0,005* | *0,158* | *0,373* | **0,446* | **0,572* |  | **0,504* | ***0,669* | *0,378* | *0,203* | *0,101* |
|  | ***SNAIL*** | | *0,180* | *0,035* | **-0,423* | *-0,210* | *0,118* | *0,355* | **0,420* | **0,504* |  | **0,422* | *-0,019* | **0,435* | *0,313* |
|  | ***FOSL1*** | | *0,379* | *0,286* | *0,030* | *0,182* | **0,469* | **0,538* | **0,494* | ***0,669* | **0,422* |  | *0,297* | *0,293* | *0,109* |
|  | ***ID1*** | | *-0,129* | *-0,193* | **0,417* | **0,545* | *0,214* | *-0,093* | *-0,156* | *0,378* | *-0,019* | *0,297* |  | *-0,016* | *-0,041* |
|  | ***POU5F1*** | | **0,408* | *0,295* | *-0,219* | *0,029* | *0,274* | **0,422* | **0,426* | *0,203* | **0,435* | *0,293* | *-0,016* |  | ***0,622* |
|  | ***NANOG*** | | *0,277* | *0,143* | *-0,217* | *-0,017* | *0,109* | *0,176* | *0,201* | *0,101* | *0,313* | *0,109* | *-0,041* | ***0,622* |  |

**Table S4A: Spearman’s correlation coefficients of 5FU fingerprint.** Spearman´s correlation coefficients (r_S_) range between -1 and 1, indicating negative and positive correlation, respectively. │r_S_│> 0,6 is considered to be a “strong” correlation (**); 0,4 < │r_S_│< 0,6 is considered to be a “weak” correlation (*); │r_S_│< 0,4 is considered to be no correlation.

|  |  | *5FU fingerprint* | | | | | | | | | | | | | |
| --- | --- | --- | --- | --- | --- | --- | --- | --- | --- | --- | --- | --- | --- | --- | --- |
| ***5FU***  ***FINGERPRINT*** |  | | ***MKI67*** | ***CCNA2*** | ***CCNB1*** | ***CCNB2*** | ***EPCAM*** | ***CDH1*** | ***CK8*** | ***CK18*** | ***SNAIL*** | ***FOSL1*** | ***ID1*** | ***POU5F1*** | ***NANOG*** |
|  | ***MKI67*** | |  | *0,000* | *0,009* | *0,018* | *0,000* | *0,000* | *0,000* | *0,006* | *0,092* | *0,000* | *0,228* | *0,000* | *0,009* |
|  | ***CCNA2*** | | *0,000* |  | *0,002* | *0,040* | *0,000* | *0,000* | *0,000* | *0,107* | *0,744* | *0,007* | *0,069* | *0,005* | *0,180* |
|  | ***CCNB1*** | | *0,009* | *0,002* |  | *0,000* | *0,000* | *0,251* | *0,021* | *0,961* | *0,000* | *0,782* | *0,000* | *0,040* | *0,041* |
|  | ***CCNB2*** | | *0,018* | *0,040* | *0,000* |  | *0,000* | *0,871* | *0,125* | *0,140* | *0,049* | *0,088* | *0,000* | *0,788* | *0,873* |
|  | ***EPCAM*** | | *0,000* | *0,000* | *0,000* | *0,000* |  | *0,000* | *0,000* | *0,000* | *0,272* | *0,000* | *0,044* | *0,009* | *0,308* |
|  | ***CDH1*** | | *0,000* | *0,000* | *0,251* | *0,871* | *0,000* |  | *0,000* | *0,000* | *0,001* | *0,000* | *0,384* | *0,000* | *0,099* |
|  | ***CK8*** | | *0,000* | *0,000* | *0,021* | *0,125* | *0,000* | *0,000* |  | *0,000* | *0,000* | *0,000* | *0,144* | *0,000* | *0,059* |
|  | ***CK18*** | | *0,006* | *0,107* | *0,961* | *0,140* | *0,000* | *0,000* | *0,000* |  | *0,000* | *0,000* | *0,000* | *0,056* | *0,348* |
|  | ***SNAIL*** | | *0,092* | *0,744* | *0,000* | *0,049* | *0,272* | *0,001* | *0,000* | *0,000* |  | *0,000* | *0,860* | *0,000* | *0,003* |
|  | ***FOSL1*** | | *0,000* | *0,007* | *0,782* | *0,088* | *0,000* | *0,000* | *0,000* | *0,000* | *0,000* |  | *0,005* | *0,005* | *0,308* |
|  | ***ID1*** | | *0,228* | *0,069* | *0,000* | *0,000* | *0,044* | *0,384* | *0,144* | *0,000* | *0,860* | *0,005* |  | *0,884* | *0,701* |
|  | ***POU5F1*** | | *0,000* | *0,005* | *0,040* | *0,788* | *0,009* | *0,000* | *0,000* | *0,056* | *0,000* | *0,005* | *0,884* |  | *0,000* |
|  | ***NANOG*** | | *0,009* | *0,180* | *0,041* | *0,873* | *0,308* | *0,099* | *0,059* | *0,348* | *0,003* | *0,308* | *0,701* | *0,000* |  |

**Table S4B: p-values for Spearman’s correlation of 5FU gene expression fingerprint.**

|  | **Cutoff – median** | | **Cutoff – quartile 1** | | **Cutoff – quartile 3** | |
| --- | --- | --- | --- | --- | --- | --- |
|  | **HR (95% CI)** | **p** | **HR (95% CI)** | **p** | **HR (95% CI)** | **p** |
|  |  |  |  |  |  |  |
| ***Prolif.*** | 3,271 (1,289 – 8,301) | ***0,013** | 1,863 (0,633 – 5,479) | 0,258 | 2,086 (0,902 – 4,821) | 0,085 |
| ***MKI67*** | 2,698 (1,109 - 6,563) | ***0,029** | 2,504 (0,744 - 8,432) | 0,138 | 3,303 (1,455 - 7,495) | ***0,004** |
| ***CCNA2*** | 1,705 (0,738 - 3,939) | 0,212 | 1,673 (0,569 - 4,919) | 0,35 | 1,980 (0,857 - 4,575) | 0,11 |
| ***CCNB1*** | 2,216 (0,939 - 5,232) | 0,069 | 1,286 (0,477 - 3,464) | 0,619 | 1,364 (0,561 - 3,317) | 0,493 |
| ***CCNB2*** | 2,034 (0,862 - 4,800) | 0,105 | 2,254 (0,670 - 7,587) | 0,189 | 1,345 (0,553 - 3,273) | 0,513 |
|  |  |  |  |  |  |  |
| ***Differen.*** | 1,776 (0,768 – 4,106) | 0,176 | 1,038 (0,409 – 2,634) | 0,938 | 2,113 (0,914 – 4,885) | 0,08 |
| ***EPCAM*** | 2,005 (0,849 - 4,732) | 0,112 | 1,624 (0,552 - 4,775) | 0,378 | 2,671 (1,169 - 6,103) | ***0,02** |
| ***CDH1*** | 2,632 (1,082 - 6,404) | ***0,033** | 1,196 (0,444 - 3,223) | 0,723 | 1,884 (0,797 - 4,451) | 0,149 |
| ***CK8*** | 1,121 (0,494 - 2,540) | 0,785 | 1,001 (0,395 - 2,540) | 0,998 | 1,347 (0,554 - 3,275) | 0,511 |
| ***CK18*** | 1,740 (0,753 - 4,021) | 0,195 | 1,257 (0,467 - 3,387) | 0,65 | 1,748 (0,741 - 4,125) | 0,202 |
|  |  |  |  |  |  |  |
| ***EMT*** | 1,340 (0,588 – 3,057) | 0,486 | 2,441 (0,725 – 8,217) | 0,15 | 1,704 (0,722 – 4,021) | 0,224 |
| ***SNAI1*** | 1,078 (0,475 - 2,443) | 0,858 | 2,471 (0,734 - 8,320) | 0,144 | 2,171 (0,938 - 5,021) | 0,07 |
| ***FOSL1*** | 2,645 (1,088 - 6,435) | ***0,032** | 1,277 (0,474 - 3,442) | 0,628 | 2,243 (0,970 - 5,188) | 0,059 |
| ***ID1*** | 1,082 (0,477 - 2,452) | 0,851 | 1,139 (0,423 - 3,068) | 0,797 | 1,051 (0,414 - 2,667) | 0,916 |
|  |  |  |  |  |  |  |
| ***Plurip.*** | 1,526 (0,660 – 3,527) | 0,323 | 0,794 (0,313 – 2,016) | 0,628 | 2,625 (1,157 – 5,955) | ***0,021** |
| ***POU5F1*** | 1,936 (0,821 - 4,568) | 0,131 | 1,138 (0,422 - 3,065) | 0,799 | 2,349 (1,029 - 5,362) | ***0,043** |
| ***NANOG*** | 1,176 (0,516 - 2,684) | 0,7 | 0,758 (0,299 - 1,927) | 0,561 | 1,597 (0,677 - 3,767) | 0,285 |

**Table S5: Univariate analysis modeling DFS using 5FU gene expression fingerprint data**

|  | **Cutoff – median** | | **Cutoff – quartile 1** | | **Cutoff – quartile 3** | |
| --- | --- | --- | --- | --- | --- | --- |
|  | **HR (95% CI)** | **p** | **HR (95% CI)** | **p** | **HR (95% CI)** | **p** |
|  |  |  |  |  |  |  |
| ***Prolif.*** | 0,831 (0,372 – 1,855) | 0,651 | 0,594 (0,254 – 1,389) | 0,23 | 0,912 (0,362 – 2,298) | 0,845 |
| ***MKI67*** | 0,668 (0,297 – 1,504) | 0,33 | 1,065 (0,398 – 2,854) | 0,9 | 0,351 (0,104 – 1,177) | 0,09 |
| ***CCNA2*** | 0,926 (0,416 – 2,062) | 0,851 | 0,968 (0,384 – 2,440) | 0,946 | 0,881 (0,350 – 2,220) | 0,788 |
| ***CCNB1*** | 1,021 (0,459 – 2,274) | 0,959 | 0,568 (0,243 – 1,327) | 0,192 | 0,931 (0,369 – 2,346) | 0,879 |
| ***CCNB2*** | 0,990 (0,445 – 2,206) | 0,981 | 0,743 (0,308 – 1,793) | 0,509 | 0,844 (0,315 – 2,261) | 0,735 |
|  |  |  |  |  |  |  |
| ***Differen.*** | 0,843 (0,387 – 1,855) | 0,678 | 0,504 (0,220 – 1,155) | 0,105 | 0,985 (0,391 – 2,483) | 0,975 |
| ***EPCAM*** | 0,864 (0,387 – 1,929) | 0,721 | 0,482 (0,211 – 1,103) | 0,084 | 0,956 (0,379 – 2,410) | 0,924 |
| ***CDH1*** | 0,567 (0,248 – 1,297) | 0,179 | 0,376 (0,166 – 0,849) | ***0,019** | 1,025 (0,407 – 2,584) | 0,958 |
| ***CK8*** | 1,311 (0,587 – 2,928) | 0,509 | 0,624 (0,267 – 1,460) | 0,277 | 1,311 (0,543 – 3,162) | 0,547 |
| ***CK18*** | 0,561 (0,245 – 1,283) | 0,171 | 0,626 (0,268 – 1,463) | 0,279 | 0,218 (0,051 – 0,926) | ***0,039** |
|  |  |  |  |  |  |  |
| ***EMT*** | 0,719 (0,320 – 1,620) | 0,427 | 1,072 (0,425 – 2,701) | 0,883 | 0,512 (0,175 – 1,499) | 0,222 |
| ***SNAI1*** | 0,891 (0,399 – 1,989) | 0,778 | 0,633 (0,271 – 1,480) | 0,292 | 0,782 (0,292 – 2,094) | 0,624 |
| ***FOSL1*** | 0,512 (0,224 – 1,717) | 0,113 | 0,735 (0,305 – 1,773) | 0,493 | 0,520 (0,178 – 1,523) | 0,233 |
| ***ID1*** | 0,850 (0,381 – 1,898) | 0,691 | 0,836 (0,347 – 2,017) | 0,691 | 0,416 (0,124 – 1,395) | 0,156 |
|  |  |  |  |  |  |  |
| ***Plurip.*** | 0,598 (0,262 – 1,368) | 0,224 | 1,000 (0,397 – 2,520) | 1 | 0,630 (0,215 – 1,845) | 0,4 |
| ***POU5F1*** | 0,752 (0,334 – 1,692) | 0,49 | 0,834 (0,346 – 2,011) | 0,685 | 0,607 (0,208 – 1,779) | 0,364 |
| ***NANOG*** | 0,885 (0,341 – 1,975) | 0,765 | 1,662 (0,568 – 1,908) | 0,354 | 0,916 (0,364 – 2,309) | 0,149 |
|  |  |  |  |  |  |  |

**Table S6: Univariate analysis modeling DFS using gene expression data in untreated PDS.**
